# CHAT-RT study: ChatGPT in radiation oncology—a survey on usage, perception, and impact among DEGRO members

**DOI:** 10.1186/s13014-025-02721-9

**Published:** 2025-09-15

**Authors:** Dinah Konnerth, Alev Altay-Langguth, Diana-Coralia Dehelean, Sebastian H. Maier, Montserrat Pazos, Paul Rogowski, Stephan Schönecker, Chukwuka Eze, Stefanie Corradini, Claus Belka, Sebastian N. Marschner

**Affiliations:** 1 Department of Radiation Oncology, LMU University Hospital, LMU Munich, Marchioninistraße 15, 81377 Munich, Germany; 2 German Cancer Consortium (DKTK), Partner Site Munich, Munich, Germany; 3 Bavarian Cancer Research Center (BZKF), Munich, Germany

**Keywords:** CHAT-GPT, Questionnaire, DEGRO, Radiation oncology, LLM

## Abstract

**Background:**

Radiation oncology is increasingly turning to Artificial Intelligence (AI) - and in particular Chat Generative pre-trained transformer (ChatGPT) - for decision support, patient education, and workflow efficiency. Despite promising gains, questions about accuracy, General Data Protection Regulation (GDPR)-compliance and ethical use persist, especially in high-stakes cancer care. To clarify real-world attitudes and practices, we surveyed members of the German Society of Radiation Oncology (DEGRO) on their use, perceptions, and concerns regarding ChatGPT across clinical, research, communication, and administrative tasks.

**Methods:**

An anonymous online survey was implemented via LimeSurvey platform and distributed to all members of the DEGRO in Germany, Austria, and Switzerland between April and June 2024. The 40-item questionnaire—covering demographics, radiotherapy experience, and ChatGPT’s clinical, research, communication, and administrative applications—was developed through a narrative literature review, ChatGPT-assisted drafting, back-translation, expert validation, and pilot testing. Fully completed responses were used for descriptive statistics and analysis.

**Results:**

Of 213 respondents, 159 fully completed the survey. Participants were predominantly based in Germany (92.5%), worked in university hospitals (74.2%), and identified as radiation oncologists (54.7%), with a broad range of radiotherapy experience (< 1 year: 7.5%; >15 years: 24.5%). Awareness of ChatGPT was high (94.9%), yet actual use varied: 32.1% never used it, while 35.2% employed it regularly for administrative tasks and 30.2% for manuscript drafting. Mid-career clinicians (6–10 years’ experience) showed the greatest enthusiasm—44% agreed it saves time and 72% planned further integration—though all career stages (71.7% overall) expressed strong interest in formal training. Satisfaction was highest for administrative (94.6%) and manuscript support (91.7%) but lower for technical queries (66.7%). Major concerns included misinformation (69.2%), erosion of critical thinking (57.9%), and data-privacy risks (57.2%).

**Conclusion:**

Our survey demonstrates high awareness and adoption of ChatGPT for administrative and educational tasks, alongside more cautious use in clinical decision-making. Widespread concerns about misinformation, critical-thinking erosion, and data privacy—especially among early- and mid-career clinicians—underscore the need for targeted AI training, rigorous validation, and transparent governance to ensure safe, effective integration into patient care.

## Background

Radiation oncology is on the edge of significant innovation driven by AI and machine learning. At the forefront is ChatGPT (OpenAI Inc., San Francisco, CA, USA), a powerful large language model trained on extensive text corpora that could support clinical decision-making, facilitate patient engagement, and advance education. Its potential goes beyond routine tasks, offering real-time guidance in complex clinical scenarios and helping break down technical information for both healthcare professionals and patients.

Recent studies have emphasized ChatGPT’s multifaceted applications and challenges across medicine, including its ability to streamline clinical workflows, expand patient education, and bolster research efforts [1–5]. However, concerns remain about reliability, hallucination, data protection, and ethical use—particularly in regions adhering to strict regulations such as the General Data Protection Regulation. In radiation oncology, precise accuracy and ethical integrity are paramount because even small errors could lead to significant risks for patients.

Within Germany, Austria, and Switzerland, navigating these challenges requires balancing AI-driven innovation with stringent legal and ethical standards. ChatGPT holds promise for simplifying patient information, promoting shared decision-making, and enhancing treatment planning. Yet questions persist about the validity of its suggestions, the potential for biased outputs, and the requirement for consistent human oversight to prevent harm.

This study focuses on the German-speaking radiation oncology community’s experiences and views on ChatGPT. Through a detailed questionnaire, we explore how professionals use, perceive, and address the benefits and conflicts associated with ChatGPT in clinical practice, research, patient communication, and administrative tasks. By capturing these insights, we aim to offer a nuanced perspective that informs broader discussions on AI integration in radiation oncology and guides safe, effective adoption strategies.

## Methods

The study’s methodology was structured to investigate ChatGPT’s usage, familiarity, benefits, risks, and ethical implications in radiation oncology. We reviewed the literature to identify gaps and key themes, then used ChatGPT to draft survey questions on demographics, work experience, and its use in clinical, research, communication, and administrative tasks - ensuring clarity and cultural relevance in both German and English through back-translation. These questions were refined through expert review, ensuring clarity, neutrality, and relevance while addressing ethical and privacy considerations.

A pilot survey was conducted with a select group of radiation oncology professionals to test the questionnaire’s clarity, usability, content relevance and to estimate average completion time and identify any technical issues. Feedback from this phase guided revisions, resulting in a finalized survey incorporating various question types such as Likert-type scale items (with balanced positive/negative wording), binary (yes/no) questions, and multiple-choice responses. Some questions also provided space for respondents to offer additional comments in free-text form to capture diverse participant experiences and perspectives (see questionnaire in the Appendix). We ensured anonymity and confidentiality to encourage honest answers, with informed consent provided on the survey’s start page.

The survey was distributed online to all 1213 DEGRO members in Germany, Austria, and Switzerland - including radiation oncologists, medical physicists, and radiation therapy technologists - via direct email and the DEGRO member mailing list. Distribution occurred over an eight-week period between April and June 2025, with two reminder emails sent to maximize response rate. It was conducted using LimeSurvey (version 5.5.0, LimeSurvey GmbH, Hamburg, Germany), a secure platform compliant with strict data-protection standards including GDPR-compliant data storage on encrypted servers. Informed consent was obtained digitally right before participation, and the anonymous survey did not require ethical approval under local regulations. Responses were collected entirely anonymously, with no IP addresses or other identifiers recorded, ensuring that individual participants could not be traced.

Only fully completed questionnaires were included in the analysis. Data were automatically exported to Microsoft Excel (Microsoft, Redmond, WA, USA) for initial cleaning and summarization using absolute numbers and percentages. Descriptive statistical analyses were also carried out using SPSS version 29 (IBM, Armonk, NY, USA).

## Results

A total of 213 participants (response rate: 17.6%) responded to the questionnaire, with 159 ( ≙ 13,1%) completing it in full. Of the 159 fully completed questionnaires analyzed, the majority of respondents were based in Germany (92.5%), with smaller proportions working in Austria (4.4%) and Switzerland (3.1%,). Over half of participants identified as radiation oncologists (54.7%), followed by those in research roles (12.6%) and medical physicists (11.3%). Most worked in university hospitals (74.2%) with clinical work as their main focus (80%), while others were in private practice (14.5%) or city hospitals (10.7%). Further details are displayed in Table 1.

**Table 1:** Participants’ characteristics

Category	Subcategory	*n*	%
Country of residence	Germany	147	92.5%
	Austria	7	4.4%
	Switzerland	5	3.1%
Professional role	Radiation Oncologist	87	54.7%
	Research Role	20	12.6%
	Medical Physicist	18	11.3%
	Other*	34	21.4%
Institution type	University Hospital	118	74.2%
	Private Practice	23	14.5%
	City Hospital	17	10.7%
Experience in radiotherapy	< 1 year	12	7.5%
	1–5 years	43	27.0%
	6–10 years	44	27.7%
	11–15 years	21	13.2%
	> 15 years	39	24.5%

*Includes RTTs, nuclear medicine, radiology, oncology, administration

Awareness of ChatGPT was high, with 94.9% reporting they had heard of it. In terms of technical familiarity, 51.6% described themselves as “moderately familiar,” 28.9% as “not familiar but willing to learn,” 15.7% as “very familiar,” and 2.5% as “extremely familiar.”

Use patterns varied: 32.1% never used ChatGPT, 28.3% used it rarely, 13.8% several times a month, 13.2% several times a week, 6.9% daily, and 5.7% multiple times per day. Beyond ChatGPT, 27.7% of respondents used other AI tools, most commonly DeepL for translation. Nearly half held a free ChatGPT 3.5 account (47.8%), while 13.2% subscribed to the paid 4.0 Pro version.

When asked about specific applications, the most frequently reported uses of ChatGPT were for administrative tasks such as drafting emails or documents (35.2%) and for creating or revising scientific manuscripts (30.2%). Other common applications included preparation of conference presentations (22.0%), development of research proposals (20.1%), and team-based project planning (17.0%). Less frequent uses were technical clarifications in radiotherapy (15.1%), data analysis and interpretation (15.7%), literature identification (14.5%), research summaries (14.5%,), student teaching materials (12.6%), staying up-to-date with technology (9.4%), and patient counseling support (7.5%).

**Fig. 1:**
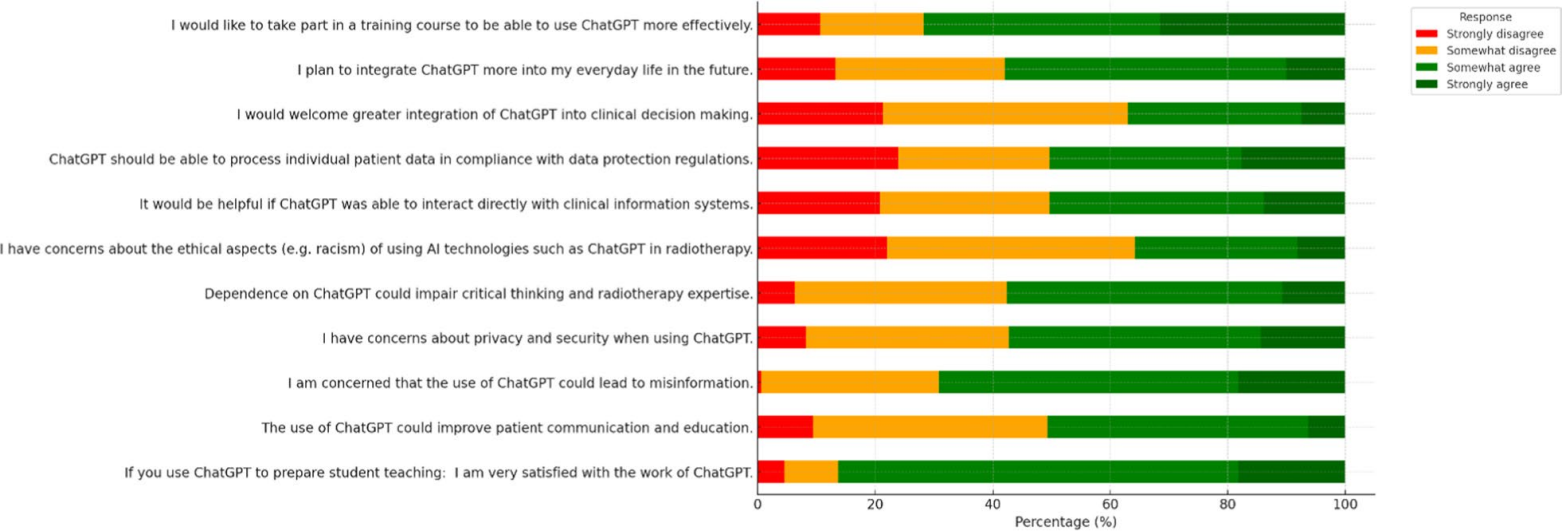
Stacked horizontal bar chart showing the distribution of agreement (strongly disagree to strongly agree) with ten key statements about ChatGPT’s role in radiotherapy. Colors correspond to response categories: red = strongly disagree, orange = somewhat disagree, light green = somewhat agree, dark green = strongly agree

Regarding perceived benefits, 47.8% of respondents felt ChatGPT helped most with rephrasing and text processing, 36.5% with creation or summarization of texts, and another 36.5% for private-matter queries. Fewer felt it was helpful for manuscript proposals (22.0%), ethics or research-application drafting (19.5%), outlining research projects (18.9%), presentations (13.2%), patient information materials (11.9%), or clinical-decision information (6.3%). One-third (33.3%) indicated that ChatGPT did not help them in any of the listed areas (Fig. 1).

**Fig. 2:**
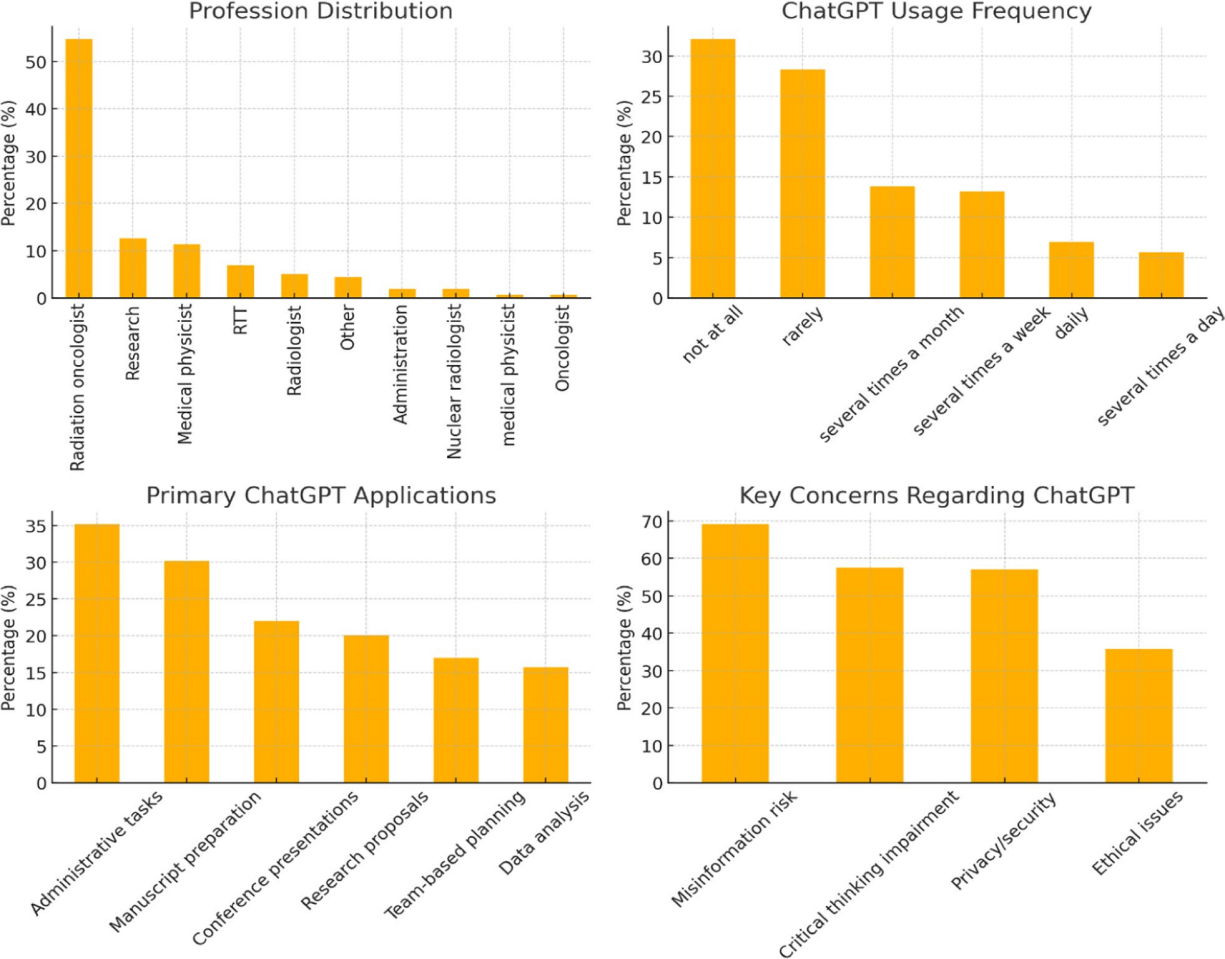
Top‑left: profession distribution; top‑right: ChatGPT usage frequency; bottom‑left: primary ChatGPT applications; bottom‑right: key concerns regarding ChatGPT

Concerns were widespread: 69.2% agreed that ChatGPT could lead to misinformation; 57.9% feared dependence might impair critical thinking and radiotherapy expertise; 57.2% had privacy or security concerns; and 35.8% were worried about ethical issues such as bias or racism (Fig. 2).

**Fig. 3:**
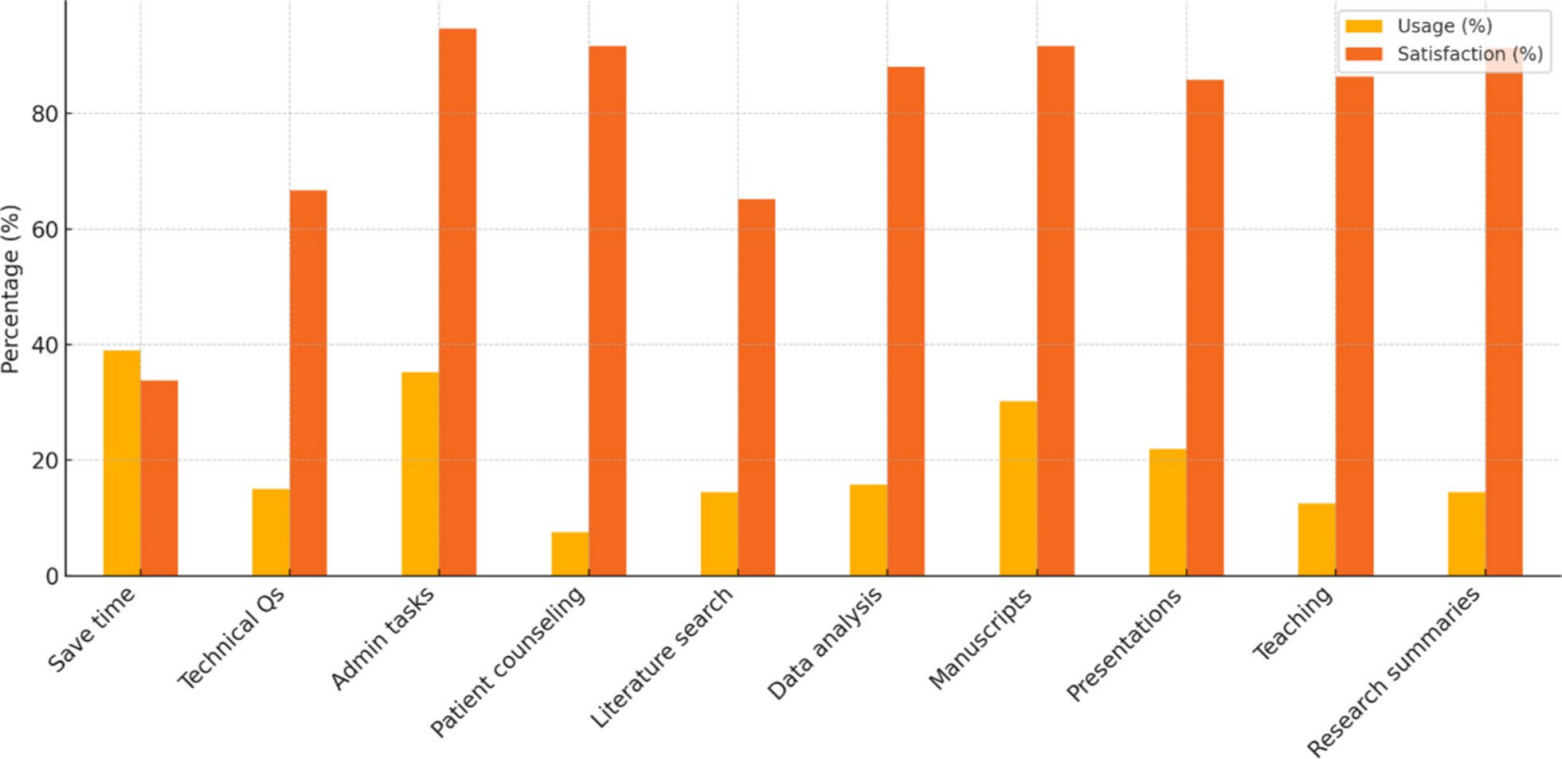
Grouped bar chart summarizing each key use-case: usage (%): percentage of all respondents who reported using ChatGPT for that task (yellow bars). Satisfaction (%): among those users, the percentage who somewhat/strongly agreed they were satisfied (orange bars)

Among those who used ChatGPT for a given task, satisfaction was generally high: 94.6% of administrative-task users (*n* = 53/56) and 91.7% of manuscript-preparation users (*n* = 44/48) reported that they agreed or strongly agreed they were satisfied with ChatGPT’s performance. Satisfaction was lower, however, among those using it for technical questions in radiotherapy (66.7%, *n* = 16/24) (Fig. 3).

Looking ahead, 57.9% planned to integrate ChatGPT more into their daily work, and 71.7% expressed interest in training to use it more effectively. About half the cohort (50.3%) considered direct integration with clinical information systems and compliant handling of patient data to be desirable, while only 37.1% welcomed deeper integration into clinical decision making.

**Fig. 4:**
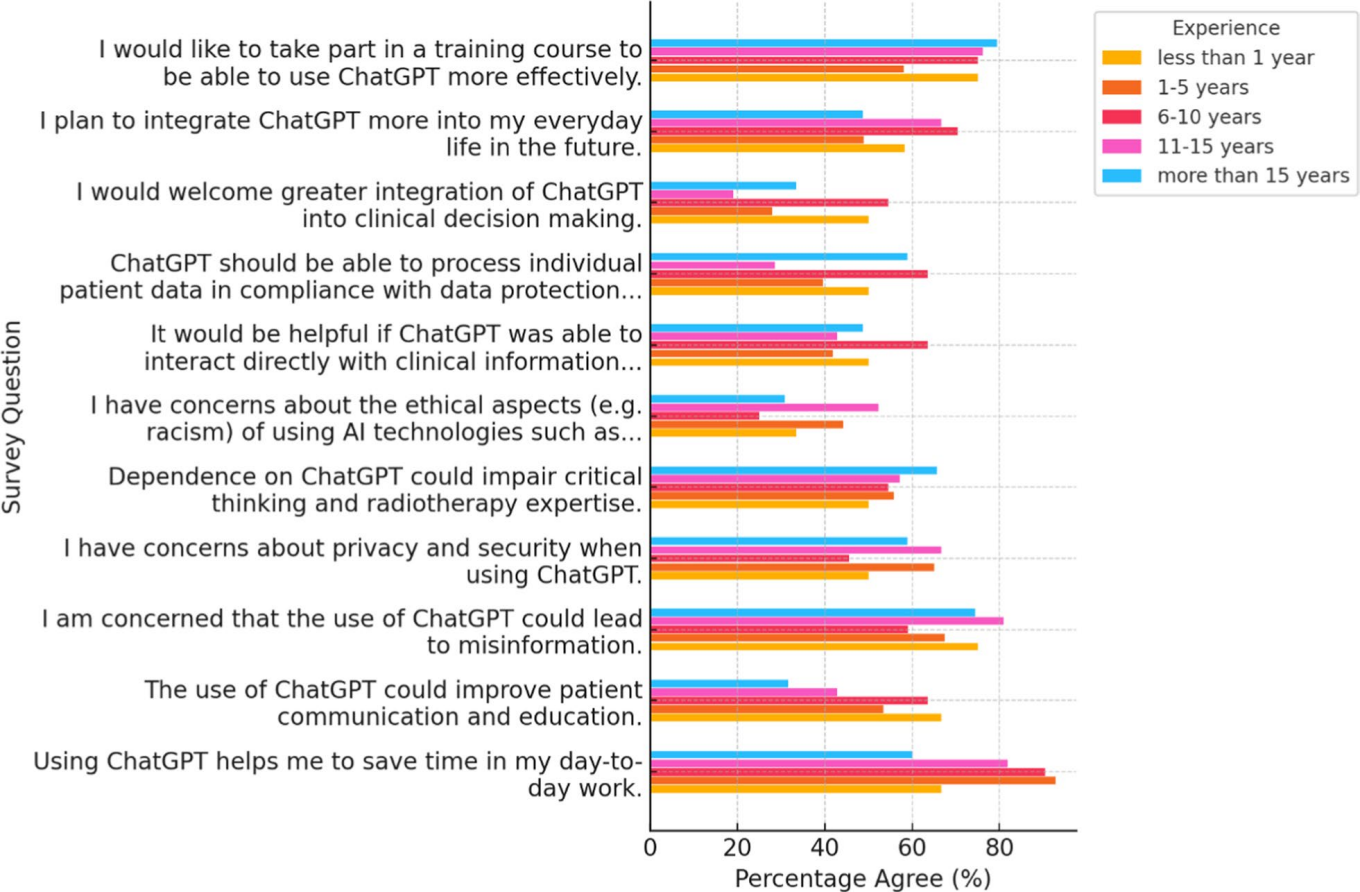
Stacked horizontal bars show the percentage of respondents who “somewhat” or “strongly” agree with each statement, stratified by experience in radiotherapy (< 1, 1–5, 6–10, 11–15, > 15 years). Each colour represents one experience group; bar lengths indicate agreement rates

When stratified by radiotherapy experience (Fig. 4), agreement that ChatGPT “helps me save time” ranged from 26% in the > 15-year cohort to 44% in the 6–10-year group, versus an overall rate of 33.8%. Plans to integrate ChatGPT into daily practice peaked at 72% among those with 6–10 years’ experience and fell to 35% in the most senior group (> 15 years), mirroring reported usage frequencies (daily/weekly use 12% vs. 6%, respectively). Interest in formal AI training was uniformly strong − 69% of > 15-year respondents through 79% of those with < 1 year expressed a desire for courses (overall 71.7%).

Mid-career professionals (6–10 years) reported the highest levels of both enthusiasm (time savings, integration plans) and apprehension (privacy, misinformation). Senior clinicians remained comparatively guarded, and early-career staff displayed the greatest appetite for training.

## Discussion

By surveying 159 radiation-oncology professionals across Germany, Austria, and Switzerland, we generated the first comprehensive map of ChatGPT’s adoption, utility, and user concerns. Our findings reveal a clear role-dependent divide: university-based radiation oncologists and researchers use ChatGPT frequently − 35.2% for administrative tasks and 30.2% for manuscript drafting - while private-practice clinicians seldom engage, reflecting differing workflow demands. Despite near-universal awareness (94.2%), one-third of respondents never used ChatGPT, and satisfaction - though high for clerical (94.6%) and writing support (91.7%) - drops markedly for technical queries (66.7%). These results suggest that, while ChatGPT excels as a writing assistant, its uptake in clinical decision-making hinges on addressing domain-specific reliability and integration challenges, particularly in non-academic settings where structured support and training may be lacking.

These results align with prior studies evaluating ChatGPT in radiation oncology. For example, Huang et al. benchmarked ChatGPT-4 on the American College of Radiology (ACR) in-training exam and Gray Zone cases, demonstrating robust performance (74.6% accuracy) but highlighting domain-specific blind spots [6]. Similarly, an exploratory study found that ChatGPT could accurately answer technical radiotherapy questions with only 63–75% accuracy, raising concerns about the consistency and reliability of ChatGPT in clinical scenarios [7]. The Green Journal reported that ChatGPT can streamline research workflows, particularly literature identification and protocol drafting, mirroring our finding that 14.5% of participants used ChatGPT for scientific publication searches, and 20.1% for proposal development, with ≈ 90% satisfaction [8]. These data suggest that while ChatGPT excels in text-centric tasks, its integration into specialized, safety-critical functions remains limited by reliability and domain expertise gaps.

Participants’ attitudes toward ChatGPT were decidedly ambivalent: while one-third (33.8%) agreed that it accelerates routine tasks, over half (57.6%) worried about its potential to undermine critical thinking. Moreover, despite widespread recognition of its utility for patient education, only 7.5% reported using ChatGPT for counselling or informational leaflets. This gap underscores the imperative to pair stringent data‐protection and accuracy controls with governance frameworks that harmonize AI‐driven efficiencies and the irreplaceable insights of clinical expertise.

Adoption is also modulated by career experience. Clinicians with < 1 year and 6–10 years in the field are the most optimistic − 44% and 33% agree that ChatGPT saves time and as many as 72% plan deeper integration - yet they simultaneously register the strongest anxieties about privacy breaches and loss of critical-thinking skills. Senior practitioners (> 15 years) remain more guarded, reflecting established workflows and higher thresholds for technological trust. This “career-stage paradox” suggests that early adopters see value but remain wary of the tool’s epistemic limits - an ambivalence that could potentially be addressed through targeted AI education. Indeed, 71.7% of all respondents expressed a desire for formal training that covers prompt engineering, output verification, and GDPR.

Respondents also expressed significant reservations about AI integration - most notably the risk of misinformation (69.2%), potential erosion of critical thinking (57.6%), and data-privacy concerns (57.2%). Although ChatGPT is readily adopted for administrative and writing tasks, these findings highlight reluctance to entrust it with clinical decision‐making, given its dependence on precise, domain‐specific knowledge and the serious patient‐safety implications of any inaccuracies.

A recent study by Guckenberger et al. [9] systematically evaluated the potential of ChatGPT to support young radiation oncology researchers. Eight clinical scientists with varying levels of experience completed seven typical scientific tasks, including literature-based work and statistical analyses, within a three-hour timeframe using ChatGPT v3.5. While less experienced participants perceived the chatbot as helpful, they did not achieve the performance level of their more experienced colleagues. Moreover, ChatGPT occasionally produced incorrect but plausible results in more complex statistical tasks, which, particularly among less experienced users, led to false assumptions. The authors concluded that ChatGPT can provide time savings and support in standardized text-based tasks but cannot replace scientific expertise, highlighting the need for critical appraisal when interpreting AI-generated content.

This finding illustrates that while ChatGPT can structure certain tasks and provide useful starting points, effective scientific work still relies on domain knowledge, methodological expertise, and critical interpretation. Without these competencies, users risk overreliance on flawed AI-generated results. Consequently, ChatGPT should be integrated as a complementary tool, with its output subject to thorough expert review.

Ethical issues overall were prominent: 35.8% of respondents highlighted algorithmic bias and fairness as key challenges, echoing broader debates in medical AI. Concerns about reliability, loss of empathy, and data security further limit ChatGPT’s perceived role in patient communication and clinical decision-making - paralleling reports from India and Taiwan, where ChatGPT is embraced for education and research but less so in direct care [10, 11].

A key strength of our study is its focus on frontline radiation oncology practitioners across both academic and non-academic settings, providing a broad perspective on ChatGPT’s utility in everyday workflows. However, our sample only included German-speaking countries, which limits global generalizability. Although the survey was distributed to all 1,213 DEGRO members, the response rate was modest (159 respondents, ≈ 13%), raising the possibility of non-response bias, as those more interested in AI may have been more likely to participate. Subgroup comparisons should also be interpreted cautiously, as small sample sizes in some strata limit representativeness. Self-selection bias may have inflated satisfaction ratings, as users predisposed to AI adoption were more likely to participate. Furthermore, we relied on self-reported measures of use and satisfaction, without objective performance metrics or qualitative follow-up to elucidate specific failure modes. Our questionnaire was restricted to predefined domains of use and concern, which may not fully capture the breadth and nuance of participants’ experiences; qualitative methods such as interviews could provide deeper insights. Lastly, our cross-sectional design precludes the assessment of evolving attitudes as newer AI models and institutional policies emerge.

Nevertheless, the reservations expressed - misinformation (69.2%), critical-thinking erosion (57.6%), data-privacy risk (57.2%), algorithmic bias (35.8%) - map directly onto actionable governance priorities. Mitigating hallucinations through better grounding and uncertainty quantification, diversifying training datasets to reduce bias, and enforcing rigorous GDPR-level protections may represent important steps if language models are to progress from administrative helper to clinical co-pilot [12–15], ensuring training datasets are representative to prevent biased outputs [16], and enforcing strict data-protection measures (e.g., GDPR compliance) [17].

Looking beyond large language models, AI holds broad promise to transform healthcare delivery and economics. Predictive analytics can flag at-risk patients earlier, potentially reducing admissions and associated costs, while precision‐medicine algorithms could tailor therapies to individual profiles, limiting ineffective treatments and adverse reactions. Automation of administrative workflows - billing, coding, claims processing, scheduling, and documentation - offers further opportunities to free clinician time for patient care, optimize resource allocation, and streamline supply chains through demand forecasting. Real‐time patient monitoring systems may avert hospitalizations by detecting early warning signs [18]. Indeed, McKinsey estimates that a comprehensive digital transformation in Germany, powered by AI and other innovations, could yield annual savings of up to €42 billion - approximately 12% o total health and care expenditures [19].

## Conclusion

In summary, radiation-therapy professionals in Germany, Austria, and Switzerland are highly aware of ChatGPT and already report using it for administrative and scholarly work. Although concerns remain around ethics, reliability, privacy, and clinical integration, strong demand for AI training and clear non-clinical benefits indicate potential benefits and interest in AI applications outside clinical decision-making. With appropriate education, validation, and governance, large language models may serve a complementary role in supporting workflows, teaching, and research activities, while ensuring that clinical expertise and judgment remain central.

## Data Availability

No datasets were generated or analysed during the current study.

## Appendix

**Table:**

Question	Answer Options
Your job description	Radiotherapist, Nuclear medicine specialist, Radiologist, Oncologist, Medical physicist, Medical technologist in radiology (MTR), Research, Administration, Other, No answer
	PhD student in medical physics, MTA/Laboratory, Nursing, Med. Documentation assistant, Study team, Study coordinator
Which country do you work in?	Germany, Austria, Switzerland, No answer
In which area of healthcare do you mainly work?	University hospital, Hospital, Private clinic, Medical practice/MVZ, Research institution, No answer
In which area are you mainly active?	Clinic, Research, Teaching, Administration, No answer
How many years of experience do you have in the field of radiotherapy?	Less than 1 year, 1–5 years, 6–10 years, 11–15 years, More than 15 years, No answer
Have you heard of ChatGPT?	Yes, No, No answer
How familiar are you with the technical aspects of ChatGPT?	Extremely familiar, Very familiar, Moderately familiar, Not familiar but willing to learn, Not interested, No answer
How often do you use ChatGPT?	Several times a day, Daily, A few times a week, A few times a month, Rarely, Not at all, No answer
Do you use other AI tools? If so, which ones?	Open-ended, No answer
I have a ChatGPT 3.5 Standard account (free).	Yes, No, No answer
I have a ChatGPT 4.0 Pro account (paid).	Yes, No, No answer
Using ChatGPT helps me save time.	I do not use ChatGPT, I use it and disagree, I use it and agree, No answer
I use ChatGPT to clarify technical questions in radiotherapy.	No, Yes—sometimes, Yes—regularly, No answer
Satisfaction with ChatGPT’s technical support.	Strongly disagree, Somewhat disagree, Somewhat agree, Strongly agree, No answer
I use ChatGPT for administrative tasks.	No, Yes—sometimes, Yes—regularly, No answer
Satisfaction with ChatGPT in administration.	Strongly disagree, Somewhat disagree, Somewhat agree, Strongly agree, No answer
I use ChatGPT for patient counseling.	No, Yes—sometimes, Yes—regularly, No answer
Satisfaction with ChatGPT in patient counseling.	Strongly disagree, Somewhat disagree, Somewhat agree, Strongly agree, No answer
I use ChatGPT to identify scientific literature.	No, Yes—sometimes, Yes—regularly, No answer
Satisfaction with ChatGPT in literature search.	Strongly disagree, Somewhat disagree, Somewhat agree, Strongly agree, No answer
I use ChatGPT for data analysis and statistics.	No, Yes—sometimes, Yes—regularly, No answer
Satisfaction with ChatGPT in data analysis.	Strongly disagree, Somewhat disagree, Somewhat agree, Strongly agree, No answer
I use ChatGPT to create or revise manuscripts.	No, Yes—sometimes, Yes—regularly, No answer
Satisfaction with manuscript support.	Strongly disagree, Somewhat disagree, Somewhat agree, Strongly agree, No answer
I use ChatGPT to prepare presentations.	No, Yes—sometimes, Yes—regularly, No answer
Satisfaction with presentation preparation.	Strongly disagree, Somewhat disagree, Somewhat agree, Strongly agree, No answer
I use ChatGPT for student teaching.	No (not involved), No (do not use it), Yes—sometimes, Yes—regularly, No answer
Satisfaction with student teaching support.	Strongly disagree, Somewhat disagree, Somewhat agree, Strongly agree, No answer
I use ChatGPT for summaries and reviews from data.	No, Yes—sometimes, Yes—regularly, No answer
Satisfaction with summaries/reviews.	Strongly disagree, Somewhat disagree, Somewhat agree, Strongly agree, No answer
ChatGPT supports my research proposals.	No, Yes—sometimes, Yes—regularly, No answer
Satisfaction with proposal development.	Strongly disagree, Somewhat disagree, Somewhat agree, Strongly agree, No answer
I use ChatGPT to stay up to date in radiotherapy.	No, Yes—sometimes, Yes—regularly, No answer
Satisfaction with staying up to date.	Strongly disagree, Somewhat disagree, Somewhat agree, Strongly agree, No answer
ChatGPT is used in my team for scientific projects.	No, Yes—sometimes, Yes—regularly, No answer
Satisfaction with team project support.	Strongly disagree, Somewhat disagree, Somewhat agree, Strongly agree, No answer
ChatGPT could improve patient communication and education.	Strongly disagree, Somewhat disagree, Somewhat agree, Strongly agree, No answer
In which areas does ChatGPT help best?	Obtaining clinical info, Outlining research, Manuscript prep, Text rephrasing, Applications, Patient info, Presentations, Summaries, Private matters, Not at all
I’m worried ChatGPT could cause misinformation.	Strongly disagree, Somewhat disagree, Somewhat agree, Strongly agree, No answer
I have privacy/security concerns with ChatGPT.	Strongly disagree, Somewhat disagree, Somewhat agree, Fully agree, No answer
ChatGPT addiction could impair critical thinking.	Strongly disagree, Somewhat disagree, Somewhat agree, Strongly agree, No answer
I have ethical concerns (e.g. racism) about AI in radiotherapy.	Strongly disagree, Somewhat disagree, Somewhat agree, Strongly agree, No answer
Helpful if ChatGPT could interact with clinical systems.	Strongly disagree, Somewhat disagree, Somewhat agree, Strongly agree, No answer
ChatGPT should process patient data (with compliance).	Strongly disagree, Somewhat disagree, Somewhat agree, Strongly agree, No answer
I would welcome ChatGPT in clinical decision making.	Strongly disagree, Somewhat disagree, Somewhat agree, Strongly agree, No answer
I plan to use ChatGPT more in the future.	Strongly disagree, Somewhat disagree, Somewhat agree, Strongly agree, No answer
I want to attend a training course on ChatGPT.	Strongly disagree, Somewhat disagree, Somewhat agree, Strongly agree, No answer

## Abbreviations

ACR: American College of Radiology
AI: Artificial Intelligence
DEGRO: Deutsche Gesellschaft für Radioonkologie e.V. (German Society of Radiation Oncology)
GDPR: General Data Protection Regulation
GPT: Generative pre-trained transformer
LLM: Large language model
RT: Radiotherapy
